# Practical Notes

**Published:** 1909-10-16

**Authors:** 


### PRACTICAL NOTES.

**To Relieve Pain of Insect Bites.**

Iodine in saponated petroleum (30 to 40 grains to the ounce) affords relief to the pain caused by insect-bites and stings. A few drops rubbed in a mosquito bite or wasp sting allay the pain instantaneously.

**Pruritus Ani**

In cases of Pruritus Ani the region must be kept scrupulously clean by washing twice daily, sponging with soap and warm water after each action of the bowels, and, after drying, applying a little boric acid powder locally. The following ointment will often relieve the symptom, especially if the application is made at night: Hydrargyri subchloridi, ʒij.; bismuthi subnitratis, ʒiss.; tincturæ aconiti, ♏viij.; glycerini, ʒij.; unguenti sambuci, ℥j. Misce. Fiat ung.—*Lockhart Mummery.*

